# Preconditioning the Initial State of Feeder-free Human Pluripotent Stem Cells Promotes Self-formation of Three-dimensional Retinal Tissue

**DOI:** 10.1038/s41598-019-55130-w

**Published:** 2019-12-12

**Authors:** Atsushi Kuwahara, Suguru Yamasaki, Michiko Mandai, Kenji Watari, Keizo Matsushita, Masayo Fujiwara, Yoriko Hori, Yasushi Hiramine, Daiki Nukaya, Miki Iwata, Akiyoshi Kishino, Masayo Takahashi, Yoshiki Sasai, Toru Kimura

**Affiliations:** 1 Regenerative & Cellular Medicine Kobe Center, Sumitomo Dainippon Pharma Co., Ltd., Chuo, Kobe, 650-0047 Japan; 2 Regenerative & Cellular Medicine Office, Sumitomo Dainippon Pharma Co., Ltd., Chuo, Kobe, 650-0047 Japan; 3 Laboratory for Retinal Regeneration, RIKEN Center for Developmental Biology, Chuo, Kobe, 650-0047 Japan; 4 Laboratory for Neurogenesis and Organogenesis, RIKEN Center for Developmental Biology, Chuo, Kobe, 650-0047 Japan

**Keywords:** Regenerative medicine, Stem-cell biotechnology, Stem-cell differentiation

## Abstract

A three-dimensional retinal tissue (3D-retina) is a promising graft source for retinal transplantation therapy. We previously demonstrated that embryonic stem cells (ESCs) can generate 3D-retina *in vitro* using a self-organizing stem cell culture technique known as SFEBq. Here we show an optimized culture method for 3D-retina generation from feeder-free human pluripotent stem cells (hPSCs). Although feeder-free hPSC-maintenance culture was suitable for cell therapy, feeder-free hPSC-derived aggregates tended to collapse during 3D-xdifferentiation culture. We found that the initial hPSC state was a key factor and that preconditioning of the hPSC state by modulating TGF-beta and Shh signaling improved self-formation of 3D-neuroepithelium. Using the preconditioning method, several feeder-free hPSC lines robustly differentiated into 3D-retina. In addition, changing preconditioning stimuli in undifferentiated hPSCs altered the proportions of neural retina and retinal pigment epithelium, important quality factors for 3D-retina. We demonstrated that the feeder-free hiPSC-derived 3D-retina differentiated into rod and cone photoreceptors *in vitro* and *in vivo*. Thus, preconditioning is a useful culture methodology for cell therapy to direct the initial hPSC state toward self-organizing 3D-neuroepithelium.

## Introduction

The retina is the main visual sensory tissue in mammals. During retinal development, the optic cup derived from the rostral diencephalon is composed of inner and outer walls that differentiate into the neural retina (NR) and retinal pigment epithelium (RPE), respectively. NR progenitors are widely present in the developing NR epithelium and function to expand to generate photoreceptors and other types of retinal neurons.

Recent advances in stem cell biology have enabled *in vitro* differentiation of retinal progenitors and their derivatives from pluripotent stem cells (PSCs)^1–23^. Moreover, mouse and human embryonic stem cell (ESC) aggregates have the ability to self-organize optic cups in three-dimensional (3D) culture^4,5^. The ESC-derived NR self-organizes the formation of multiple retinal layers reminiscent of the postnatal retina. NR progenitors in this culture system have a radial glia-like epithelial morphology, and expand to give rise to photoreceptors and other retinal neurons in a stage-dependent manner, resembling the process *in vivo*^4,5^. The self-organizing stem cell culture technique developed in these studies, named SFEBq (serum-free floating culture of embryoid body-like aggregates with quick aggregation), is now used to generate 3D-organoids as an attractive model for recapitulating mouse and human organogenesis *in vitro*^24–26^.

Toward future applications in cell therapy, we previously reported a modified SFEBq culture method for robust generation of 3D-retina^6^. In our method, designated the BMP method, timed addition of bone morphogenetic protein 4 (BMP4) to the 3D-differentiation culture selectively induces human retinal progenitors at the expense of telencephalic progenitors. Furthermore, we showed that hESC-derived retinal progenitors can be induced to differentiate into NR-RPE-conjugated two-domain 3D-retina, another type of retinal cup, using a fate-biasing culture technique named the induction-reversal culture method. The two-domain 3D-retina contains two key features: a ciliary margin-like stem cell niche and a centro-peripherally polarized NR. The human ciliary margin-like stem cell niche, self-formed at the margin of the NR and RPE, fosters a retinal stem-like cell population and functions to expand the NR by de novo progenitor generation. The centro-peripherally polarized NR tends to maintain a stratified continuous epithelium structure, reminiscent of the developing NR epithelium *in vivo*. Therefore, using the BMP method combined with the induction-reversal culture method, hESC-aggregates can be robustly differentiated into two-domain 3D-retina that self-forms a high-quality stratified continuous NR epithelium^6^.

Retinitis pigmentosa is a group of hereditary diseases characterized by a loss of photoreceptors and is the major cause of untreatable blindness in developed countries. Transplantation of photoreceptors and/or retinal progenitors is a promising therapeutic option^27–31^. Indeed, transplantation of retinal tissues and cells from primary embryonic tissues has the potential to improve visual function^32–34^. To supply adequate amounts of retinal tissues and cells for cell therapy, 3D-retina generated from hPSCs is a promising graft source. Photoreceptor precursors purified from ESC-derived retinal tissue can survive and make contact with the host retina^8,9,14,19,35,36^. Retinal sheet dissected from mouse ESC-3D-retina can engraft into even severely degenerated mouse retina, and graft-derived photoreceptors form direct synaptic connections with the host bipolar cells^37^. In addition, mouse iPSC-derived 3D-retina transplanted into end-stage retinal degeneration model mice (*rd1*) improved visual function^29^. Human 3D-retina, generated from hESCs using the BMP method and the induction-reversal culture method, differentiated into rod and cone photoreceptors that developed a structured outer nuclear layer in a primate model of severe retinal degeneration^38^. Importantly, we recently published another paper about functional maturation of human 3D-retina transplantation in the end-stage retinal degeneration model mice^39^. These studies demonstrate the potential of hPSC-derived 3D-retina as a graft source for retinal transplantation therapy.

In our previous studies, hESCs used for retinal differentiation were routinely maintained on a feeder layer of mitotically-inactivated mouse embryonic fibroblasts (MEFs)^5,6^, based on published protocols^40,41^. However, coculture with feeder cells is not a preferred procedure for generating clinical-grade human 3D-retina, partly because of the high costs. Therefore, we aimed to differentiate 3D-retina from feeder-free hESCs and hiPSCs. Application of Rho-associated kinase (ROCK) inhibitor to hPSCs markedly prevents dissociation-induced apoptosis and enhances survival to expand dissociated hPSCs^42^. Recombinant laminin-511 E8 fragments (LM511-E8), containing minimal integrin-binding sites, have strong adhesive activity for hPSCs^43^. Nakagawa *et al*. developed a feeder-free culture method to reprogram and expand hPSCs using a culture medium named StemFit combined with ROCK inhibitor and LM511-E8 matrix^44^.

Toward retinal cell therapy, we aimed to generate 3D-retina from hPSCs maintained in a feeder-free (Ff-) culture condition. Here we established an efficient differentiation culture technique to direct Ff-hPSCs toward self-organizing 3D-retina formation by modulating TGF-beta and Shh signaling pathways. The TGF-beta superfamily signaling pathway, including TGF-beta, Nodal/Activin and BMP signaling pathways, has negative effects on neural differentiation in early development; therefore, inhibition of TGF-beta signaling pathways utilizes to promotes neural differentiation in PSC-differentiation culture^2,45^. Shh signaling promotes ventralization and posteriorization in neural patterning and retinal tissue formation in hESC-differentiation culture in some context^5^. Using this new culture method, we demonstrated that several hESC and hiPSC lines robustly differentiated into 3D-retina. Furthermore, we transplanted human iPSC-derived 3D-retina to examine whether feeder-free iPSC-derived 3D-retina can engraft and undergo maturation *in vivo*.

## Methods

### Ff-hPSC maintenance culture

hESC-KhES-1 line (*Rx::*Venus line) was used according to the hESC research guidelines of the Japanese government and cultured at RIKEN. hiPSC-1231A3 line was established by Kyoto University and derived from ePBMC(R) purchased from Cellular Technology Limited (http://www.immunospot.com/), and gifted from Kyoto University^44^. hiPSC-C3 and hiPSC-LPF11 lines were a gift from N. Takamura, M. Higuchi, and A. Fujiki (Sumitomo Dainippon Pharma). hPSCs were maintained on LM511-E8 matrix (Nippi) in StemFit medium (Ajinomoto) according to a published protocol^44^ with slight modifications. Prior to passaging, culture plates (Iwaki) were coated with LM511-E8 matrix in PBS at 37 °C for at least 1 h. hPSC colonies were treated with TrypLE Select Enzyme (Gibco) at 37 °C for 4–7 min, and dissociated into single cells by gentle pipetting. The dissociated PSCs were suspended in StemFit medium and counted. The single-cell PSC suspension was plated on LM511-E8 matrix-coated plates at a density of 700–1700 cells/cm^2^ and cultured in StemFit medium with 10 µM Y-27632^42^. The medium was changed to StemFit medium without Y-27632 on day 1 and further changed on days 3, 5, and 6 at least. Passages were performed every sixth or seventh day.

### Retinal differentiation from Ff-hPSCs by the preconditioning, d0-SAG, and BMP methods

**Figure 1:**
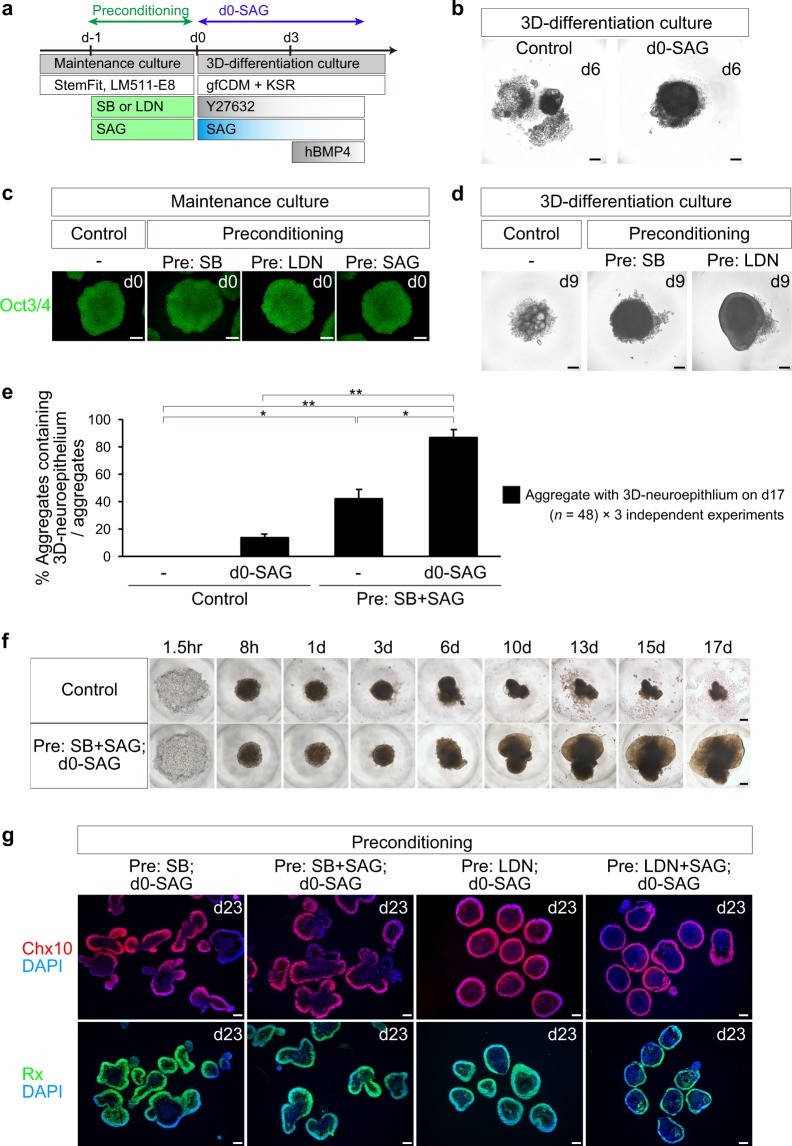
Preconditioning and d0-SAG treatment promote self-formation of 3D-neuroepithelium from Ff-hiPSCs. **(a)** Timing for preconditioning of Ff-hiPSCs (Preconditioning) and SAG-treatment on day 0 (d0-SAG). (**b**) Bright-field view of Ff-hiPSC-1231A3-derived aggregates and controls on day 6. Note that SAG treatment on day 0 (d0-SAG) improves aggregate growth compared with untreated controls (Control). (**c**) Immunostaining for Oct3/4 (green) in preconditioned Ff-hiPSC-1231A3 cells and control cells on day 0. (**d**) Bright-field view of preconditioned Ff-hiPSC-1231A3-derived aggregates and controls on day 9. Note that preconditioning with SB and LDN improves aggregate growth compared with untreated controls (Control). (**e**) Percentages of aggregates with a neuroepithelium on day 17 derived from preconditioned Ff-hiPSC-1231A3 cells and untreated controls in combination with or without d0-SAG treatment. Data represent mean ± SEM of three independent experiments (each experiment with 48 aggregates; counted aggregates with neuroepithelium, Control = 0, d0-SAG = 20, Pre: SB + SAG = 61, Pre: SB + SAG plus d0-SAG = 124. *p < 0.05. **p < 0.01. ANOVA followed by post-hoc Tukey’s test. (**f**) Time-lapse imaging of a SB + SAG-preconditioned-Ff-hiPSC-derived aggregate and a control. (**g**) Immunostaining of preconditioned-Ff-hiPSC-derived aggregates on day 23. Red, Chx10 (upper panels). Green, Rx (lower panels). Blue, nuclear staining with DAPI. Scale bars represent 200 µm in (**b,c,d,f,g**). Similar results were obtained in three independent experiments.

For the preconditioning method, hPSC colonies in StemFit medium were treated with, 5 µM SB431542 (SB), 100 nM LDN193189 (LDN), and/or 300 nM smoothened agonist (SAG) for 18–30 h prior to differentiation. The SFEBq culture method was then performed as described^6^. hPSC colonies were treated with TrypLE Select Enzyme at 37 °C for 4–7 min, and dissociated into single cells by gentle pipetting. The dissociated iPSCs were quickly reaggregated using low-cell-adhesion 96-well plates with V-bottomed conical wells (Sumilon PrimeSurface plates; Sumitomo Bakelite) in differentiation medium (100 µl; 10,000 cells/well) supplemented with 20 µM Y-27632 and 30 nM SAG (d0-SAG method) under 5% CO_2_ at 37 °C. The differentiation medium was growth factor-free CDM (gfCDM) supplemented with 10% KSR, while gfCDM alone comprised 45% Iscove’s modified Dulbecco’s medium (IMDM; Gibco), 45% Hams F12 (F12; Gibco), Glutamax, 1% chemically defined lipid concentrate (Gibco), and 450 µM monothioglycerol (Sigma)^46^. The day of SFEBq culture initiation was defined as day 0, and recombinant human BMP4 (R&D Systems) was added at 1.5 nM (55 ng/ml) on day 3. Thereafter, the concentration of BMP4 was diluted by half with a half-medium change every third day. For the Ff-hiPSC without preconditioning, treatment with 300 nM on day 0 efficiently improved aggregate growth (d0-SAG method, Fig. 1b). By using the preconditioning, d0-SAG and BMP methods, the expression of Rx and Chx10 was induced and maintained at least on day 14–23.

### Long-term NR culture by the induction-reversal culture method

Induction-reversal culture and long-term NR culture were performed as described^6^. The NR epithelium generated from hPSCs was subjected to two-step induction-reversal culture under the following conditions. For the induction culture, NR-containing aggregates generated from hPSCs on days 14–23 were transferred from 96-well plates to 9-cm non-cell-adhesive petri dishes (Sumitomo Bakelite; approximately 32 aggregates/9-cm dish), and further cultured in suspension for 3 days under 5% CO_2_ in DMEM/F12-Glutamax medium (Gibco) containing 1% N2 supplement (Gibco), 3 µM CHIR99021 (GSK3-inhibitor; Stemgent), and 5 µM SU5402 (FGFR-inhibitor; Sigma). For the reversal culture, the floating aggregates with an RPE-like thin epithelium were cultured in suspension under 5% CO_2_ in NR-differentiation medium containing DMEM/F12-Glutamax medium, 1% N2 supplement, 10% FBS (GE Healthcare HyClone), 0.5 µM retinoic acid (all-trans retinoic acid, Sigma), 0.1 mM taurine (Sigma), 0.25 µg/ml Fungizone (Gibco), 100 U/ml penicillin, and 100 µg/ml streptomycin. The medium was changed every third or fourth day until day 178. Floating aggregates were analyzed using inverted microscopes (Keyence BIOREVO; Nikon Eclipse-Ti; Olympus IX83).

### Transplantation

In all animal experiments, the animals were treated in accordance with the Association for Research in Vision and Ophthalmology Statement for the Use of Animals in Ophthalmic and Vision Research. All animal experiments were conducted with approval from the Animal Research Committee at RIKEN Center for Developmental Biology Institute and only performed at RIKEN. Transplantation into the subretinal space of rats were performed as described^38,47^. SD-Foxn1 Tg(S334ter)3Lav nude rats (RD-nude rats) were obtained from the Rat Resource and Research Center^48,49^. Ff-hiPSC-derived 3D-retina (1231A3) on day 58 were transplanted to 6–8-week-old RD-nude rats. Similar results were obtained in four eyes.

### Immunohistochemistry

Immunohistochemistry was performed as described^6^. Aggregates were fixed with 4% PFA and sectioned with a cryostat (Leica). Frozen sections were treated with or without heat-based antigen retrieval in Target Retrieval solution (Dako) at 105 °C for 15 min. The primary antibodies used in this study were as follows: Chx10 (sheep; 1:500; Exalpha), Rx (guinea pig; 1:2000; Takara), Pax6 (mouse; 1:1000; BD Biosciences), Mitf (mouse; 1:500; Exalpha), Crx (rabbit; 1:200; Takara), Recoverin (rabbit; 1:1000; Proteintech), NRL (goat; 1:500; R&D Systems), RXR-gamma (rabbit; 1:500; Spring Bioscience), Rhodopsin (mouse; 1:1000; Sigma; RET-P1), S-opsin (rabbit; 1:500; Millipore), L/M-opsin (rabbit; 1:500; Millipore), PKCalpha (rabbit; 1:500; Sigma), HuNu (Ms; 1:1000; Millipore), Calbindin (rabbit; 1:500; Millipore), N-cadherin (mouse; 1:500; BD Biosciences), FoxG1 (rabbit; 1:2000; Takara), Ctip2 (rat; 1:500; abcam), Tbr1 (rabbit; 1:500; abcam). Nuclear counter-staining was performed with DAPI (Nacalai). Stained sections were analyzed with a fluorescence microscope (BIOREVO; Keyence) and a confocal microscope (FV1200; Olympus). Image analyses were performed with ImageJ 1.48 v software (NIH).

### mRNA expression analysis

Feeder-free hiPSCs were cultured in 6 well plate (~2 × 10^6^ cells) in control or preconditioning conditions (*n* = 4) and lysed with RLT buffer (QIAGEN). After differentiation, 16–32 aggregates per each time points were lysed with RLT buffer (*n* = 4). Total RNA was extracted with an RNeasy micro kit (QIAGEN) according to manufacturer’s instructions. Quantitative PCR reactions (qPCR) were performed by using TaqMan gene expression assays (Thermo Fisher Scientific). Relative mRNA expression was determined by the delta-delta Ct method with gapdh as an endogenous control. The standard error of the mean (SEM) were calculated using Excel software (Microsoft). The qPCR primers used in this study were as follows (TaqMan Probes; Thermo Fisher Scientific): gapdh (Hs02758991_g1), ACTB (Hs01060665-g1), Rx (Rax; Hs00429459_m1), Chx10 (Vsx2; Hs01584047_m1), Pax6 (Hs00240871_m1), Sox1 (Hs01057642-s1), Sox2 (Hs01053049_s1), Oct3/4 (POU5F1; Hs04260367_gH), Nodal (Hs00415443-m1), ID1 (Hs03676575-s1), Gli1 (Hs00171790_m1), Patched-1 (PTCH1; Hs00181117-m1), Gata4 (Hs00171403-m1), Gata6 (Hs00232018-m1), Hand1 (Hs02330376-s1).

### Statistical analysis

Statistical analyses were performed with Excel software (Microsoft) and R software (ver 3.6.1). Statistical significance was tested with Student’s *t*-test (parametric) for two-group comparisons and with ANOVA followed by post-hoc Tukey’s test for multi-group comparisons. Values of *p* < 0.05 were considered to indicate statistical significance. *p < 0.05; **p < 0.01 and ***p < 0.001 for all figures.

## Results

### Preconditioning of hiPSC state in feeder-free maintenance culture by modulating TGF-beta and Shh signaling promotes Self-formation of 3D-retina

Toward retinal cell therapy, we aimed to generate 3D-retina from hiPSCs maintained in a feeder-free culture condition (Ff-hiPSCs). The 1231A3 hiPSC line, established in Kyoto University using episomal vectors expressing Yamanaka factors, was maintained on LM511-E8 matrix in StemFit medium according to published protocol^44^. hiPSC-1231A3 cells expanded stably in the feeder-free maintenance culture, and their doubling time was approximately 12–18 h. 3D-retinal differentiation culture was then carried out using the SFEBq-BMP method^6^. Ff-hiPSC-1231A3 cells were dissociated into single cells, plated in V-bottom 96-well plates, and cultured in differentiation medium (gfCDM + KSR) in the presence of Y-27632. Unexpectedly, we observed that Ff-hiPSC-derived aggregates reproducibly collapsed at around day 6 in 3D-differentiation culture (Fig. 1a,b). When, by chance, we added feeder cells (mitotically-inactivated MEFs) together with dissociated Ff-hiPSCs on day 0, we found that the added MEFs improved aggregate growth in 3D-differentiation culture (Fig. S1a,b). We then found that addition of MEF-conditioned medium in 3D-differentiation culture on day 0 slightly improved aggregate growth (data not shown). These observations suggest that Ff-hiPSCs still have a potential to differentiate into 3D-neural tissue as well as hPSC on feeder cells and that the initial state of Ff-hiPSC around day 0 might be a key factor. We then reasoned that the initial state of Ff-hiPSCs may be different from that of hPSCs on feeder cells and hypothesized that addition of certain signaling molecules to the culture medium around day 0 may bias the initial state of Ff-hiPSCs toward a state suitable for 3D-differentiation culture. We designated this methodology the preconditioning method.

To test our hypothesis, we added several candidate signaling molecules around day 0 of 3D-differentiation culture and examined their effects. First, we observed that transient treatment of Ff-hiPSC-aggregates with SAG (Shh signaling agonist; starting at 300 nM on day 0 with half-dilution every third day) promoted aggregate growth during 3D-differentiation culture (Fig. 1a,b, d0-SAG). The timing of the addition was important, because SAG treatment from day 3 showed smaller effects on aggregate growth than SAG treatment from day 0.

Second, we examined the effects of preconditioning before starting 3D-differentiation culture. Ff-hiPSCs were cultured in the Ff-maintenance culture condition with FGF2 in the presence or absence of various signaling molecules for 1 day (Fig. 1a). Oct3/4 expression was not so much affected by 1 day of exposure to SB (inhibitor of TGF-beta/Nodal/Activin signaling), LDN (inhibitor of BMP signaling), and SAG (Fig. 1c, Preconditioning). Next, preconditioned Ff-hiPSCs on day 0 were dissociated into single cells, plated in V-bottom 96-well plates, and subjected to 3D-differentiation culture (Fig. 1a). The preconditioned Ff-hiPSCs formed aggregates that grew well and self-formed a neuroepithelium on day 9, unlike the case for control cells (Fig. 1d). These findings indicate that preconditioning of Ff-hiPSCs with TGF-beta/Nodal signaling inhibitor (SB) and BMP signaling inhibitor (LDN) can promote aggregate growth during 3D-differentiation culture.

We optimized the method by combining the d0-SAG treatment (Fig. 1b) and the preconditioning method (Fig. 1d). Preconditioning of Ff-hiPSCs with SB plus SAG (SB + SAG) promoted 3D-neuroepithelium self-formation in 3D-differentiation culture on day 17 (~40% of aggregates, Fig. 1e). Importantly, the d0-SAG treatment (starting at 30 nM on day 0 with half-dilution every third day) in SB + SAG-preconditioned Ff-hiPSCs enhanced aggregate growth and self-formation of 3D-neuroepithelium (~90% of aggregates, Fig. 1e). Time-lapse experiments indicated that control-iPSCs formed aggregates on day 1 and collapsed on day 6, while SB + SAG-preconditioned-iPSCs formed aggregates on day 1 and did not collapse but self-formed a neuroepithelium on day 10 (Fig. 1f). These findings suggest that the SB + SAG-preconditioning method in combination with the d0-SAG treatment may not affect initial formation of aggregates on days 1–3, but can promote aggregate growth and neuroepithelium self-formation during days 3–10.

We examined whether preconditioned Ff-hiPSC-derived neuroepithelium contained retinal progenitors. Ff-hiPSCs in maintenance culture were preconditioned for 1 day with SB, SB + SAG, LDN, and LDN plus SAG (LDN + SAG) or left untreated as control and differentiated in the presence of SAG and Y-27632 (Fig. 1a). The Ff-hiPSC-aggregates were then treated with BMP4 on day 3 and cultured in gfCDM + KSR medium. In this culture, control Ff-hiPSC-derived aggregates without preconditioning collapsed. On day 23, preconditioned-iPSC derived aggregates were fixed and immunostained for retinal progenitor markers Chx10 (also called Vsx2) and Rx (also called Rax)^50–52^. The preconditioned-iPSC-derived aggregates in each condition were found to contain a neuroepithelium on their surface, and the majority of the cells in the neuroepithelium were positive for Chx10 and Rx (Fig. 1g). Thus, hiPSCs in feeder-free maintenance culture can differentiate into a 3D-NR epithelium by using the preconditioning method and d0-SAG treatment combined with the BMP method.

Importantly, we observed that the morphology of the NR epithelium differed depending on the preconditioning conditions (Fig. 1g). Specifically, LDN-preconditioned Ff-hiPSCs (or LDN + SAG-preconditioned Ff-hiPSCs) differentiated into an NR epithelium in round-shaped aggregates, while SB-preconditioned Ff-hiPSCs (or SB + SAG-preconditioned Ff-hiPSCs) differentiated into a cup-like NR epithelium in tangentially-polarized two-domain aggregates. Among multiple preconditioning methods, we mainly used preconditioning with SB + SAG in combination with d0-SAG, because two-domain 3D-retina tend to maintain laminated stratified NR epithelium in the long term differentiation culture^6^.

### Preconditioning method improves self-formation of 3D-neuroepithelium from hESC and hiPSC Lines

Next, we investigated whether the preconditioning method is a generally useful methodology for the generation of 3D-neuroepithelium from feeder-free hESCs and hiPSCs. For this, we examined the effects of preconditioning in: 1) multiple ESC and iPSC lines; 2) another commonly used feeder-free culture medium; 3) various types of well-plates in 3D-differentiation culture; and 4) formation of non-retinal neuroepithelium.

**Figure 2:**
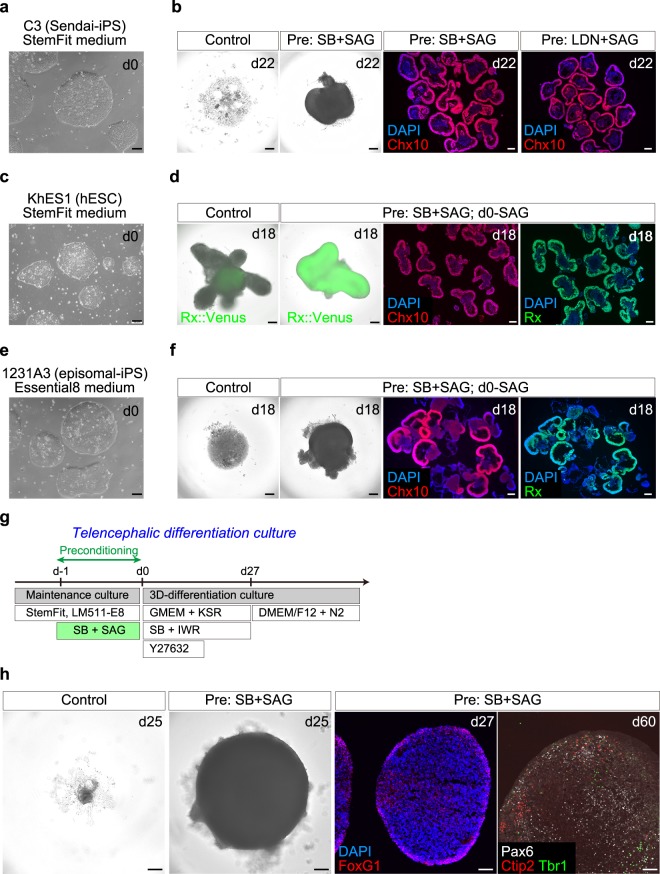
Preconditioning promotes self-formation of 3D-retina from multiple hiPSC and hESC lines in various conditions. (**a,b**) Comparison of NR epithelium-inducing efficiency between preconditioned cells and untreated controls using the hiPSC-C3 line established with Sendai virus vectors. (**a**) Bright-field view of hiPSC-C3 cells in feeder-free culture on day 0. (**b**) Bright-field view (left) and immunostaining for Chx10 (right, red) and nuclear staining with DAPI (right, blue) of aggregates on day 22. (**c,d**) Comparison of NR epithelium-inducing efficiency between preconditioned cells and untreated controls using the hESC-KhES-1 line harboring a Venus-knock-in reporter for Rx (*Rx*::Venus line). (**c**) Bright-field view of hESC-KhES-1 cells on day 0. (**d**) Bright-field view with expression of *Rx::*Venus of aggregates (green, left), and immunostaining for Chx10 (left, red) and Rx (right, green) nuclear staining with DAPI (blue) of aggregates on day 18. **(e,f**) Comparison of NR epithelium-inducing efficiency between preconditioned cells and untreated controls using the hiPSC-1231A3 line cultured on LM511-E8 matrix in Essential8 medium. (**e**) Bright-field view of hiPSCs cultured in Essential8 medium. (**f**) Bright-field view (left) and immunostaining for Chx10 (left, red) and Rx (right, green) and nuclear staining with DAPI (blue) of aggregates on day 18. **(g**,**h**) Comparison of telencephalic tissue-inducing efficiency between preconditioned cells and untreated controls using the hiPSC-1231A3 line. (**g**) Scheme of differentiation culture. (**h**) Left: Bright-field view of controls and SB + SAG-preconditioned iPSC-derived aggregates. Right: Preconditioned aggregates immunostained for FoxG1 (red) and nuclear staining with DAPI (blue) on day 27 (left), and immunostained for Pax6 (white), Ctip2 (red), and Tbr1 (green) on day 60 (right). Scale bars represent 100 µm (**a,c,e**) and 200 µm (**b,d,f,h**).

We cultured multiple ESC and iPSC lines in the preconditioning or control conditions, and induced their differentiation with the SFEBq-BMP method (Fig. 1a). As another PSC line, the hiPSC-C3 line established using Sendai virus vectors was maintained on LM511-E8 matrix in StemFit medium. hiPSC-C3 cells expanded well in the feeder-free culture condition (Fig. 2a). When dissociated control-Ff-hiPSC-C3 cells were cultured in the 3D-differentiation culture condition, the aggregates tended to collapse (Fig. 2b). In contrast, SB + SAG-preconditioned Ff-hiPSC-C3 cells cultured in the 3D-differentiation culture condition using the BMP method self-formed an NR epithelium that was positive for Chx10 on day 22 (Fig. 2b). Preconditioning of Ff-hiPSC-C3 cells with LDN + SAG also improved self-formation of an NR epithelium (Fig. 2b). Thus, preconditioning can promote self-formation of an NR epithelium from hiPSC-C3 cells established using Sendai virus vectors in the feeder-free culture condition.

We further examined the effects of preconditioning using the hESC-KhES-1 line harboring a Venus-knock-in reporter for Rx^5^(Fig. 2c). Although we previously maintained hESC-KhES-1 cells on an MEF feeder layer to develop the BMP method^6^, aggregates derived from feeder-free hESC-KhES-1 cells did not grow well, similar to the case for Ff-hiPSCs (Fig. 2d). By combining the preconditioning method with the BMP method, we found that Ff-hESC-KhES-1 cells in 3D-differentiation culture self-formed an NR epithelium expressed *Rx*::Venus (Fig. 2d). Most of Venus^+^ epithelium was positive for Chx10 (Fig. 2d). The preconditioning method with SB + SAG is therefore applicable to hESC and hiPSC lines.

We also investigated another commonly used feeder-free culture medium, Essential8^53^. Ff-hiPSC-1231A3 cells maintained in Essential8 medium on LM511-E8 matrix and cultured in the 3D-differentiation condition failed to form a neuroepithelium (Fig. 2e,f), consistent with the results in StemFit medium. By combining the preconditioning method with the BMP method, we found that Ff-hiPSCs cultured in Essential8 medium differentiated into retinal progenitors positive for Chx10 and Rx (Fig. 2f). These findings suggest that the preconditioning method is applicable to both StemFit medium and Essential8 medium.

We examined whether the effects of preconditioning were dependent on specific types of well-plates in 3D-differentiation culture. We observed that control-Ff-hiPSC-derived aggregates tended to collapse in V-bottom plates, U-bottom plates, and flat-bottom culture dishes, while preconditioned-Ff-hiPSC-derived aggregates grew well and self-formed a neuroepithelium in these three types of well-plates (Fig. S2).

As the preconditioning method significantly improved self-formation of a neuroepithelium in our 3D-retina differentiation culture, we examined its application to the generation of other neural tissue, such as telencephalic tissue. We performed 3D-differentiation culture for the induction of telencephalic tissue based on a published protocol^54^ (Fig. 2g). Control Ff-hiPSC-derived aggregate cultured in GMEM-based differentiation medium containing KSR, SB, and IWR (inhibitor of Wnt-beta-catenin signaling pathway) tended to collapse, similar to the case for 3D-retina differentiation in gfCDM + KSR medium. We found that preconditioning of Ff-hiPSCs with SB + SAG substantially improved self-formation of a neuroepithelium containing FoxG1-positive telencephalon-like cells on day 27 and layer-specific cortical neuronal markers Tbr1 and Ctip2-positive cells on day 60 (Fig. 2h).

Collectively, the above findings demonstrate that preconditioning of Ff-hPSCs with SB + SAG is a useful methodology to inhibit the collapse of Ff-PSC-derived aggregates and promote self-formation of 3D-neuroepithelium such as 3D-retina in SFEBq culture.

### mRNA expression in preconditioned hiPSCs and differentiated cells

**Figure 3:**
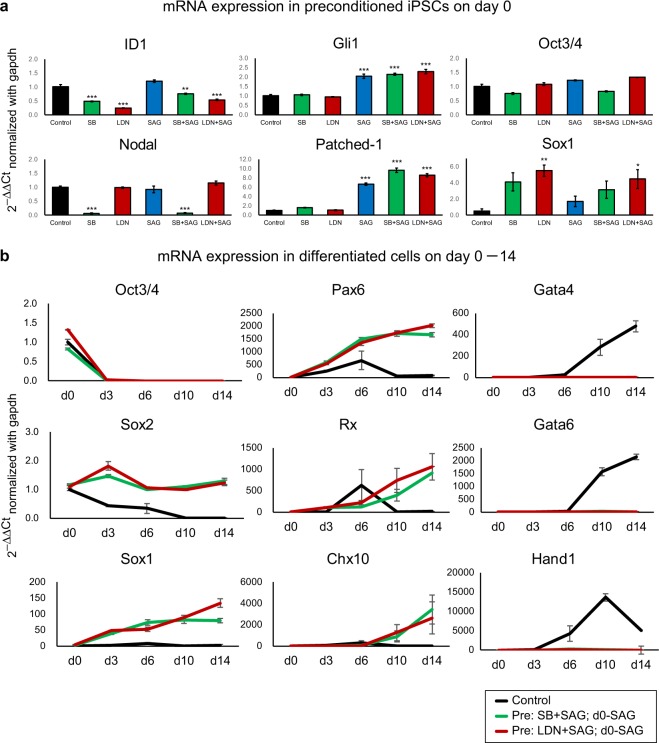
mRNA expression in preconditioned iPSCs and differentiated cells. **(a)** Ff-hiPSCs (LPF11 line established with Sendai virus vectors) were treated with SB, LDN, SAG, SB + SAG and LDN + SAG for 24 h (preconditioned iPSCs) or untreated as control (Control). mRNA levels were determined by qPCR analysis. Data are represented as mean ± SEM (*n* = 4 experiments). *p < 0.05. **p < 0.01. ***p < 0.001. ANOVA followed by post-hoc Tukey’s test. (**b**) Ff-hiPSCs (LPF11 line) were treated with SB + SAG (green line) and LDN + SAG (red line) for 24 h or untreated as control (black line). Control hiPSCs were differentiated with using the BMP method without d0-SAG method. Preconditioned hiPSCs were differentiated with using both the d0-SAG and BMP methods. Cells were lysed on day 0, 3, 6, 10 and 14 after differentiation. mRNA levels were determined by qPCR analysis. Data are represented as mean ± SEM (*n* = 4 experiments).

In the preconditioning method, a 1-day treatment with SB, LDN, SAG, SB + SAG and LDN + SAG in Ff-PSC-maintenance culture significantly improved aggregate growth and self-formation of a neuroepithelium. To examine the underlying mechanism, we tested mRNA expression in preconditioned iPSCs. Ff-hiPSC-LPF11 (Sendai) cells maintained in StemFit medium on LM511-E8 matrix were treated with SB, LDN, SAG, SB + SAG or LDN + SAG for 24 h or left untreated as controls. Total RNA extracted from the iPSCs was subjected to qPCR analysis (Figs. 3a and S3, *n = *4). First, we checked the mRNA expression of ID1 (downstream target of TGF-beta/Nodal/Activin/BMP signaling), Nodal (downstream target of TGF-beta/Nodal/Activin signaling), Gli1 and Patched-1 (downstream targets of Shh signaling)^55,56^, and confirmed that SB, LDN, SB + SAG and LDN + SAG treatment decreased the mRNA level of ID1, while SAG, SB + SAG and LDN + SAG treatment increased the mRNA levels of Gli1 and Patched-1 (Fig. 3a). These results indicate that the levels of signaling pathways in Ff-hiPSCs were affected by preconditioning method. The mRNA level of PSC marker Oct3/4 was not so much affected by preconditioning. In contrast, we found that SB, LDN, SB + SAG and LDN + SAG treatment tended to increase the mRNA level of early neural differentiation marker Sox1^57^. The increase of Sox1 was consistent with promoting effect on neural tissue formation by the preconditioning method.

We further investigated the effects of preconditioning after starting differentiation. Ff-hiPSC-LPF11 cells maintained in StemFit medium on LM511-E8 matrix were treated with SB + SAG or LDN + SAG for 24 h or left untreated as control. Then these cells were differentiated by using SFEBq, d0-SAG and BMP methods (Fig. 1A). Total RNA extracted from the differentiated cells on day 0, 3, 6, 10 and 14 was subjected to qPCR analysis (*n* = 4, 16–32 aggregates per each conditions). We tested the mRNA expression of PSCs (Oct3/4 and Sox2), neural (Sox2, Sox1, Pax6, Rx and Chx10), retinal (Pax6, Rx and Chx10) and mesoendodermal (Gata4, Gata6 and Hand1^58^) markers and normalized with gapdh mRNA expression (Fig. 3b). The mRNA level of PSC marker Oct3/4 was decreased on day 3 after differentiation in both control (without preconditioning), SB + SAG- and LDN + SAG-preconditioned cells. The mRNA level of PSC and neural marker Sox2 was decreased on day 3 in control cells (black line), whereas maintained in SB + SAG- and LDN + SAG-preconditioned cells (green and red lines, respectively). In addition, the mRNA levels of neural marker Sox1, Pax6, Rx and Chx10 did not so much increased in control on day 10 and 14, whereas highly increased in SB + SAG and LDN + SAG-preconditioned cells. Importantly, the mRNA levels of mesoendodermal marker Gata4, Gata6 and Hand1 were highly increased in control (black line) on day 10 and 14. These results suggest that SB + SAG- and LDN + SAG-preconditioned cells differentiated into neural cells, whereas control cells (without preconditioning) did not sufficiently differentiate into neural cells but tended to differentiate into mesoendodermal fate. Thus, our model is that preconditioning with SB + SAG and LDN + SAG affected the levels of signaling pathways of Ff-hPSCs and biased the Ff-hPSC state suitable for neural differentiation.

### Preconditioning of hiPSCs affects the proportions of NR and RPE

**Figure 4:**
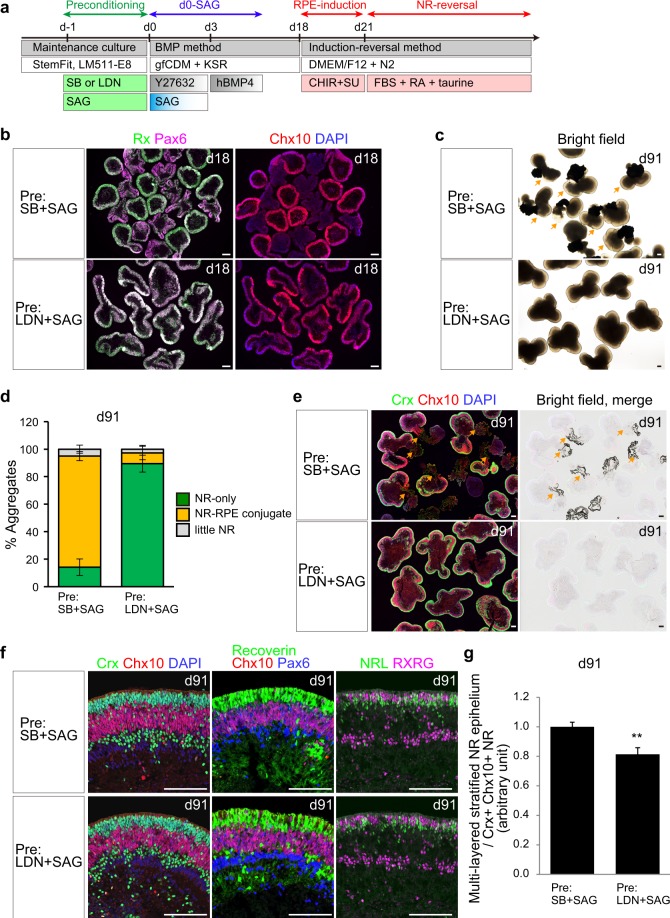
Preconditioning of Ff-hiPSCs affects the proportions of NR and RPE. Comparison of 3D-retina from hiPSCs preconditioned with SB + SAG or LDN + SAG. Ff-hiPSCs (LPF11 line) were preconditioned with SB + SAG or LDN + SAG for 24 h and then differentiated into 3D-retina using the preconditioning, BMP, and induction-reversal methods. **(a)** Scheme. (**b**) Immunostaining for retinal markers in aggregates on day 18. Immunostaining for Rx (green, left), Pax6 (purple, left), and Chx10 (red, right) and nuclear staining with DAPI (blue, right) are shown. (**c**) Bright-field view of aggregates on day 91. Note that SB + SAG-preconditioned Ff-hiPSCs-aggregates tend to form NR-RPE-conjugated two-domain aggregates. Orange arrows represent NR-RPE-conjugated two-domain aggregates. (**d**) Percentages of the three types of aggregates on day 91. Orange bars represent NR-RPE-conjugated two-domain aggregates. Data are represented as mean ± SEM (*n* = 4 experiments, with 12 aggregates per experiment). (**e**) Immunostaining for retinal markers (left) and merged bright-field view (right) of frozen sections of aggregates on day 91. Immunostaining for Crx (green) and Chx10 (red) and staining with DAPI (blue) are shown. Note that the pigmented cells in the bright-field view are the RPE. Orange arrows represent NR-RPE-conjugated two-domain aggregates. (**f**) Comparison of NR epithelium on day 91 derived from cells preconditioned with SB + SAG or LDN + SAG. Immunostaining for retinal markers and nuclear staining with DAPI in aggregates on day 91 are shown. Left: Crx (green), Chx10 (red), and DAPI (blue). Middle: Recoverin (green), Chx10 (red), and Pax6 (blue). Right: NRL (green) and RXRG (purple). (**g**) Comparison of the proportions of multilayered stratified NR epithelium on day 91 derived from cells preconditioned with SB + SAG or LDN + SAG. Data are represented as mean ± SEM (*n* = 10 aggregates). **p < 0.01. Student’s *t*-test. Similar results were obtained in three independent experiments. Scale bars represent 200 µm in (**b,c,e**) and 100 µm (**f**).

We newly developed the preconditioning method combined with the BMP method to self-form an NR epithelium from Ff-hiPSCs (Fig. 4a). We then compared 3D-retina derived with different preconditioning stimuli: pre-LDN and pre-SB. We observed that the morphology of the NR epithelium differed depending on the preconditioning conditions (Fig. 1g). To confirm the proportion of NR epithelium in the aggregates, we performed immunostaining. LDN + SAG-preconditioned Ff-hiPSC-LPF11 cells were differentiated using the BMP method and formed NR-dominant aggregates with an Rx^+^/Pax6^+^/Chx10^+^ NR epithelium on day 18 (Fig. 4b, LDN-type aggregates). In contrast, SB + SAG-preconditioned Ff-hiPSC-LPF11 cells formed two-domain aggregates containing both an Rx^+^/Pax6^+^/Chx10^+^ NR epithelium and Rx^dim^/Pax6^++^/Chx10^−^ tissue on day 18 (Fig. 4b, SB-type aggregates).

We compared the differentiation potentials of the two types of aggregates in long-term culture. For this, we cultured LDN-type aggregates and SB-type aggregates using a fate-biasing induction-reversal culture method^6^(Fig. 4a). Ff-hiPSC-derived aggregates were cultured with CHIR99021 (Wnt agonist) and SU5402 (FGFR inhibitor) for 3 days to bias the cells toward the RPE fate and then further cultured in NR-reversal culture medium containing serum, retinoic acid, and taurine for 70 days. On day 91, almost 90% of LDN-type aggregates had formed an NR epithelium on their surface and contained little pigmented RPE tissue (Fig. 4c–e). In contrast, almost 80% of SB-type aggregates had formed NR-RPE-conjugated two-domain aggregates on day 91 (Figs. 4c–e and S4a,b). Similar results were obtained using the other Ff-hiPSC line 1231A3 (Fig. S4c). These observations suggest that LDN-preconditioned hiPSCs and SB-preconditioned hiPSCs tend to differentiate into NR-dominant aggregates and NR-RPE-conjugated two-domain aggregates, respectively.

Next, we confirmed whether LDN-type aggregates and SB-type aggregates can differentiate into photoreceptors and self-form a multilayered NR epithelium. Both types of aggregates were fixed and immunostained. We found that the NR epithelium in both types of aggregates formed a photoreceptor layer with Crx^+^, Recoverin^+^, NRL^+^, and RXRG^+^ cells on its outer surface (Fig. 4e,f). In addition, an NR progenitor layer with Chx10^+^/Pax6^+^ cells was formed in the middle of the NR epithelium, and a neuronal layer with Chx10^−^/Pax6^++^ cells was formed on the inner side of the aggregates. These findings demonstrate that LDN-type aggregates and SB-type aggregates can both differentiate into photoreceptors and self-form a multilayered stratified NR epithelium. Interestingly, we found that both types of aggregates contained rosette structures in addition to a stratified continuous NR epithelium (Fig. S4d). The proportion of the stratified continuous NR epithelium was slightly higher in the SB-type aggregates (Fig. 4g), consistent with our previous report suggesting that NR-RPE-conjugated two-domain aggregates tended to maintain a continuous NR epithelium^6^.

Taken together, the above findings suggest that: 1) preconditioning of undifferentiated hiPSCs affects the proportions of NR and RPE in 3D-retina; 2) LDN-preconditioned hiPSCs and SB-preconditioned hiPSCs tend to differentiate into NR-dominant aggregates and NR-RPE-conjugated two-domain aggregates, respectively; and 3) SB-type aggregates and LDN-type aggregates can both self-form a multilayered stratified NR epithelium with an NR progenitor layer and a photoreceptor layer.

### Ff-hiPSC-derived 3D-retina differentiates into rod and cone photoreceptors *in vitro* and *in vivo*

**Figure 5:**
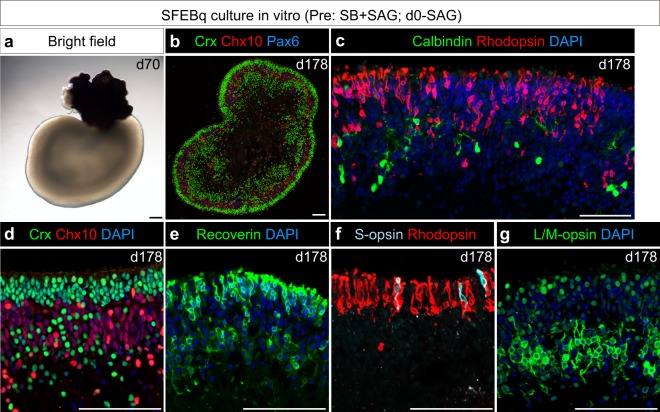
Preconditioned Ff-hiPSCs self-form a multilayered NR epithelium and differentiate into rod and cone photoreceptors. Ff-hiPSC-1231A3 cells were preconditioned with Pre: SB + SAG, treated with d0-SAG and differentiated into 3D-retina. (**a**) Bright-field view of NR-RPE-conjugated two domain aggregate (turnip-shaped) on day 70 derived from SB + SAG-preconditioned Ff-hiPSC-1231A3 cells. **(b–g**) Immunostaining for retinal markers and nuclear staining with DAPI in SB + SAG-preconditioned Ff-hiPSC-1231A3-derived 3D-retina on day 178. (**b**) Crx (green), Chx10 (red), and Pax6 (blue). (**c**) Calbindin (green), Rhodopsin (red), and DAPI (blue). (**d**) Crx (green), Chx10 (red), and DAPI (blue). (**e**) Recoverin (green) and DAPI (blue). (**f**) Rhodopsin (red) and S-opsin (light blue). (**g**) L/M-opsin (green) and DAPI (blue). Scale bars represent 100 µm in all panels.

We investigated whether hiPSCs cultured in feeder-free culture can differentiate into rod and cone photoreceptors. We differentiated Ff-hiPSC-1231A3 cells into SB-type 3D-retina (Figs. 4a and 5a; Pre: SB + SAG; d0-SAG), followed by further culture *in vitro* for 178 days as reported previously^6^. SB-type 3D-retina was fixed and immunostained for photoreceptor markers. We found that the NR epithelium in the 3D-retina self-formed a multilayered structure comprising an outer photoreceptor layer with Crx^+^ and Recoverin^+^ cells, a middle layer with Chx10^+^ cells, and an inner layer with Calbindin^+^ horizontal cells and Chx10^−^/Pax6^++^ cells (Fig. 5b–e). Importantly, the NR epithelium in the 3D-retina contained Rhodopsin^+^ rods, S-opsin^+^ cones, and L/M-opsin^+^ cones (Fig. 5c,f,g). These findings suggest that preconditioned Ff-hiPSCs have the ability to self-form a multilayered NR epithelium with a photoreceptor layer of rods and cones, similar to the case for hESCs on MEF feeder cells.

**Figure 6:**
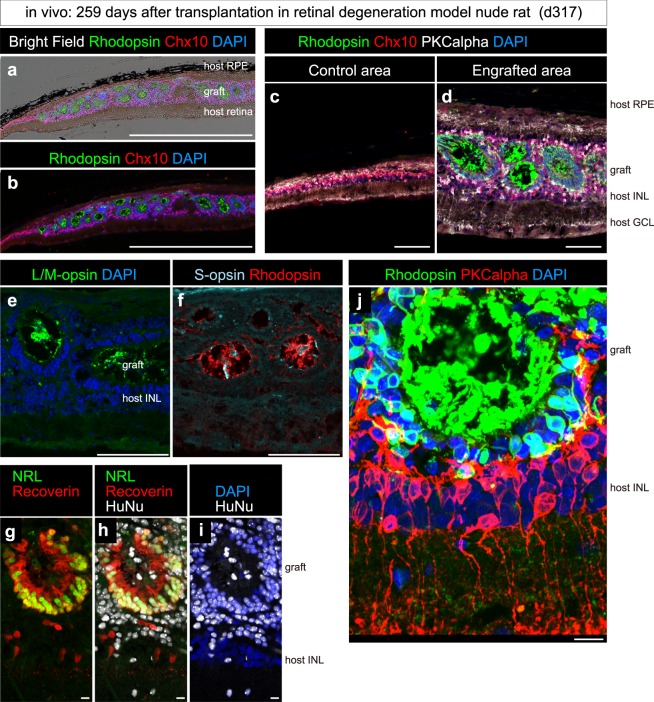
Transplantation of Ff-hiPSC-derived 3D-retina into RD-nude rats. Immunostaining of rat eyes transplanted with hiPSC-derived 3D-retina. Ff-hiPSC-1231A3 cells were preconditioned with Pre: SB + SAG, treated with d0-SAG and differentiated into 3D-retina. 3D-retina on day 58 was dissected and transplanted into the subretinal space of RD-nude rats. The rat retinas were fixed at 259 days after transplantation (317 days after initiation of differentiation). (**a**) Merged image of bright-field view and immunostaining data shown in (**b**). (**b–j**) Immunostaining for retinal markers and nuclear staining with DAPI. (**b**) Rhodopsin (green), Chx10 (red), and DAPI (blue). (**c,d**) Rhodopsin (green), Chx10 (red), PKCalpha (white), and DAPI (blue). (**e**) L/M-opsin (green) and DAPI (blue). (**f**) Rhodopsin (red) and S-opsin (light blue). (**g–i**) NRL (green), Recoverin (red), HuNu (white), and DAPI (blue). (**j**) Maximum projection image of Z-stacks stained for Rhodopsin (green), PKCalpha (red), and DAPI (blue). Note that Rhodopsin^+^ rods are in direct contact with PKCalpha^+^ bipolar cells, which have axons toward the basal side of the host retina. Similar results were obtained in four eyes. Scale bars represent 1000 µm in (**a,b**), 100 µm in (**c–f**), and 10 µm in (**g–j**). INL, inner nuclear layer; GCL, ganglion cell layer.

Toward retinal cell transplantation therapy, we examined whether preconditioned Ff-hiPSC-derived 3D-retina can engraft and undergo maturation *in vivo*. For this, we used end-stage retinal degeneration model nude rats (RD-nude rats) carrying a mutated human rhodopsin transgene (SD-Foxn1 Tg(S334ter)3-LavRrrc line)^48,49^. Ff-hiPSC-derived SB-type 3D-retina (1231A3) on day 58 was dissected and transplanted into the subretinal space of RD-nude rats, as described previously^38^. At 259 days after transplantation (317 days after initiation of differentiation), the eye balls were fixed and immunostained. We observed that the hiPSC-derived 3D-retina became engrafted between the host RPE and host NR (Fig. 6a,b). In the RD-nude rats, the host photoreceptor cells were almost completely degenerated (Fig. 6c). In the engrafted area, Rhodopsin^+^ photoreceptors formed photoreceptor rosettes (Fig. 6d) that contained Rhodopsin^+^ rods, L/M-opsin^+^ cones, and S-opsin^+^ cones with mature photoreceptor-like morphology (Fig. 6e,f and supplemental movie). Furthermore, NRL^+^/Recoverin^+^ photoreceptors in the rosettes were positive for human nuclear antigen HuNu (Fig. 6g–i). Importantly, some photoreceptors in the rosettes were in direct contact with the bipolar cell layer (Fig. 6j and supplemental movie). Collectively, these observations indicate that a preconditioned Ff-hiPSC-derived 3D-retina has the potential to engraft into the subretinal space of RD-nude rats, undergo differentiation, mature into rods and cones *in vivo*, and make direct contact with the bipolar cell layer.

## Discussion

A milestone in the realization of retinal cell transplantation therapy is the ability to supply adequate amounts of high-quality retinal tissues. Sasai and colleagues developed the SFEBq method to generate 3D-retina from hPSCs. We applied the SFEBq method to multiple types of hESCs and hiPSCs cultured in feeder-free culture conditions and unexpectedly found that Ff-PSC-derived aggregates tended to collapse during SFEBq culture. In the present study, we found that the initial state of hPSCs is a key factor, and that preconditioning of the hPSC state by modulating the levels of TGF-beta and Shh signaling can promote self-formation of 3D-neuroepithelium, including 3D-retina. The preconditioning method also provides a strategy to regulate the proportions of NR and RPE, important quality factors for a 3D-retina. We further demonstrated that Ff-hiPSC-derived 3D-retina has the potential to differentiate into rods and cones and engrafted in end-stage retinal degeneration model rats at least for 8 months. This modified SFEBq technology would provide a new way to generate adequate numbers of high quality 3D-retina suitable for cell transplantation therapy.

### Feeder-free culture and preconditioning method

Since human ESC lines were established by Thomson *et al*. (1998), human PSCs have been routinely cultured on feeder cells in many laboratories^40,41,59,60^. We previously developed a 3D-retina differentiation culture method using hESCs on feeder cells^5,6^. Mass production and quality control of feeder cell banks are associated with high costs, approximately similar to those of hPSC banks. For example, feeder cells such as MEFs are xeno-materials and must undergo virus negativity tests. Furthermore, culture of hPSCs on feeder cells requires good manipulation skills. In contrast, feeder-free culture on LM511-E8 matrix has a low cost and is easy to manipulate for hPSC expansion^43,44^. Therefore, we developed a preconditioning method to generate 3D-retina from Ff-PSCs. The combination of feeder-free PSC-culture and the modified SFEBq method is a technically simple method for generating 3D-retina, compared with our previous method.

One of the methodological challenges in 3D-differentiation culture is how to reduce the variety of organoids at both the intra-batch level and inter-batch level. Eiraku *et al*. developed the SFEBq culture method to regulate the size and quality of individual PSC-aggregates^24^. In the modified method developed in the present study, the variety of organoid quality, such as morphologies of individual organoids and proportions of NR and RPE, may be smaller than those in our previous method^6^ (for example, the percentages of NR-RPE conjugated two-domain aggregates were ~40% in the previous study and ~80% in this study). Possible mechanisms for this are that: 1) feeder-free culture may reduce the variety of PSC states compared with on-feeder culture; and 2) the preconditioning method modify the levels of signaling pathways to reduce the variety of 3D-retina. By using a combination of the Ff-PSC culture, SFEBq, preconditioning, BMP, and induction-reversal methods, it is now possible to robustly generate 3D-retina with controlled NR-RPE proportions.

The reason why feeder-free hPSC-derived aggregates in SFEBq culture tend to collapse still remain elusive. Ff-hiPSCs without preconditioning might have a possibility to prone to mesoendodermal fate instead of ectodermal fate (Fig. 3). Interestingly, Meyer and colleges developed retinal differentiation culture protocol from Ff-hiPSC: feeder-free hPSC colonies cultured in mTeSR1 + matrigel and Nutristem + Synthemax are detached to form hPSC aggregates and spontaneously differentiated into neural tissue in the absence of FGF2^61^. Ff-hiPSC maintenance culture and/or initial steps of differentiation culture are critical steps to generate 3D-retina robustly.

Previous studies reported changes made in the early steps of retinal differentiation culture: TGF-beta/Nodal signaling inhibitor^2,7^, Wnt signaling inhibitor^2,5,7^, IGF1^7,16^, ROCK inhibitor in hPSC suspension culture^42^, SFEBq method^4–6^, 2D/3D-combination culture method of hPSC aggregates^11,12^, low concentration KSR^4^, extracellular matrix addition on PSC aggregates^4,5^, 2D-conflent culture method^18^, COCO^62^ and BMP signaling agonist^6^. In the present study, we identified Shh signaling agonist in the early step promoted aggregate growth and neural tissue formation (d0-SAG method). Although Wnt priming method in mouse ESC maintenance culture was reported^63^, major improvements in human PSC retinal differentiation culture protocols were made after the removal of FGF2 to start differentiation. The removal of FGF2 greatly changes the hPSC state, therefore we developed preconditioning method in the presence of FGF2. The preconditioning method a minor modification in the maintenance culture is useful culture methodology to adjust differentiation protocol for each hPSC cell lines without major changes after starting differentiation.

The mechanisms how preconditioning (before differentiation) affected 3D-neuroepithelium formation (after differentiation) still remain unclear. Preconditioning method might consist of two steps: first, preconditioning in the presence of FGF2 changed the initial state of Ff-hPSCs in maintenance culture; then, preconditioned Ff-hPSCs efficiently self-formed 3D-neuroepithelium in differentiation culture.Multiple preconditioning methods, namely short-term treatment with SB, LDN, SAG, SB + SAG and LDN + SAG, promoted self-formation of neuroepithelium, respectively. To elucidate the state of preconditioned Ff-hPSCs, we examined mRNA expression of preconditioned Ff-hiPSCs. We found that SB- and LDN-treatments reduced the mRNA level of TGF-beta signaling target ID1 and SAG-treatment increased that of Shh signaling targets Gli1 and Patched-1. These results raise a possibility that preconditioning might bias from highly stable pluripotency-maintained state to a state suitable for 3D-differentiation culture in the levels of signaling pathways. Preconditioning with SB, LDN, SB + SAG and LDN + SAG increased the mRNA level of early neural differentiation marker Sox1. The Nodal/Activin signaling pathway promotes induction of the mesoendodermal fate, while the TGF-beta and BMP signaling pathways promote induction of the mesoendodermal fate and extraembryonic fate in the early stage and epidermal fate at the later ectodermal stage^24,25^. Indeed, control Ff-iPSC (without preconditioning) tended to differentiate into the mesoendoderm-biased state, which the mRNA levels of Sox2, Sox1, Pax6 and Chx10 were not sufficiently induced and the mRNA levels of Gata4, Gata6 and Hand1 were increased. It is therefore possible that preconditioning with the TGF-beta/Nodal/BMP signaling inhibitor and/or Shh signaling agonist may bias Ff-hPSC state to neural fate instead of mesoendodermal fate.

Importantly, preconditioning of Ff-hPSCs can control the proportions of NR and RPE within 3D-retina: LDN-preconditioned hiPSCs and SB-preconditioned hiPSCs tended to differentiate into NR-dominant aggregates and NR-RPE-conjugated two-domain aggregates, respectively. We reasoned that preconditioning might affect the positional information and local patterning of self-formed neuroepithelium along the centro-peripheral axis in the eye field.

Taken together, we believe that preconditioning might bias the initial PSC state to affect differentiation in several steps: 1) biasing from highly stable pluripotency-maintained state toward a state suitable for 3D-differentiation culture in the levels of signaling pathways, 2) promoting 3D-neuroepithelium formation and growth after differentiation and 3) affecting the local patterning within the 3D-neuroepithelium. We therefore proposed that optimization of preconditioning stimuli is the useful methodology to direct initial Ff-PSC state toward self-organizing 3D-neuroepithelium with controlled positional information.

### Future applications of preconditioned Ff-hiPSC-derived 3D-retina

Self-organizing culture of 3D-organoids is a powerful tool to study organogenesis^25,26^. A series of studies by Sasai, Eiraku and colleagues revealed the mechanism for optic cup formation^4,5,64,65^. Self-organizing hPSC culture also applied to analyze the mechanism of de novo neurogenesis from the ciliary margin-like retinal stem cell niche^6^. Canto-Soler and colleagues demonstrated that hiPSC-derived 3D-retina can respond to light stimuli^12^. The feeder-free culture and modified SFEBq method in the present study, in combination with genome editing technology, will provide a technically easy method to induce genetically-modified 3D-retina for research on organogenesis. Important issues, such as species differences in retinal development and features of human retina, key determinants for differentiation of human rods and cones, and generation of fovea structures, may be resolved in future studies.

iPSC technology brings a new dimension to drug discovery and regenerative medicine^60,66^. Modeling of inherited retinal diseases is an important theme for developing therapeutic strategies^28,67^. Ff-hiPSC-derived 3D-retina provides a platform for research on retinal diseases, such as age-related macular degeneration (AMD), retinitis pigmentosa (RP), and glaucoma^11^. Toward the development of therapies for glaucoma, PSC-derived retinal ganglion cells from 3D-retina can be cultured *in vitro* for examination of neurite outgrowth^68–70^. Primary retinal ganglion cells purified from the eye were engrafted into the retina *in vivo*^71^, raising the possibility that hPSC-3D-retina may contribute to cell therapies for glaucoma.

Clinical scientists and stem cell biologists have been developing new strategies for cell therapy. Indeed, removal of neovascular lesion followed by transplantation of an autologous iPSC-derived RPE sheet improved foveal thickness in a patient with neovascular AMD^72^. Two different teams published important studies related with RPE transplantation in patients^73,74^. In retinal degeneration diseases, such as severe AMD and RP, progressive photoreceptor loss leads to irreversible vision loss. Transplantation of human PSC-derived photoreceptors is a promising therapeutic option^27–29,75,76^. Nevertheless, numerous points remain to be determined, such as transplantation of immature cells or mature cells, retinal progenitors or photoreceptors, cell suspension or retinal sheet, unfrozen or frozen cells, contributions of material transfer, retinal state of patients (hosts), immunosuppression method, surgical devices, and manufacturing procedures compliant with cGMP. Among these, a series of studies by Takahashi, Mandai and colleagues have indicated that PSC-derived 3D-retina has the potential to engraft, undergo maturation *in vivo*, and provide 3D-retina-derived photoreceptors in direct contact with the host bipolar cell layer in retinal degeneration model animals^29,37–39^. In the present study, we observed that Ff-hiPSC-derived 3D-retina can engraft into retina *in vivo*, similar to the case with 3D-retina derived from hESCs on MEF feeder cells. We recently published another manuscript about the positive effects of human Ff-hPSC-derived 3D-retina transplantation in model animals^47^. Toward photoreceptor transplantation therapy, we are now addressing the manufacturing process and a new methodology to control the quality of 3D-retina using SFEBq technology.

### Highlights

• Optimized self-organizing culture can generate 3D-retina from feeder-free hPSCs

• hPSC preconditioning by modulating TGF-beta and Shh promotes 3D-retina formation

• hPSC preconditioning affects the proportions of neural retina and RPE

• hPSC-3D-retina differentiates into rod and cone photoreceptors *in vitro* and *in vivo*

## Supplementary information

Supplementary Figures

## Data Availability

The datasets generated during the current study are not publicly available due to commercialisation related to research findings but are available from the corresponding author on reasonable request.
