# Publisher Correction: Preconditioning the Initial State of Feeder-free Human Pluripotent Stem Cells Promotes Self-formation of Three-dimensional Retinal Tissue

**DOI:** 10.1038/s41598-020-58892-w

**Published:** 2020-02-05

**Authors:** Atsushi Kuwahara, Suguru Yamasaki, Michiko Mandai, Kenji Watari, Keizo Matsushita, Masayo Fujiwara, Yoriko Hori, Yasushi Hiramine, Daiki Nukaya, Miki Iwata, Akiyoshi Kishino, Masayo Takahashi, Yoshiki Sasai, Toru Kimura

**Affiliations:** 1 Regenerative & Cellular Medicine Kobe Center, Sumitomo Dainippon Pharma Co., Ltd., Chuo, Kobe 650-0047 Japan; 2 Regenerative & Cellular Medicine Office, Sumitomo Dainippon Pharma Co., Ltd., Chuo, Kobe 650-0047 Japan; 3 Laboratory for Retinal Regeneration, RIKEN Center for Developmental Biology, Chuo, Kobe 650-0047 Japan; 4 Laboratory for Neurogenesis and Organogenesis, RIKEN Center for Developmental Biology, Chuo, Kobe 650-0047 Japan

Correction to: *Scientific Reports* 10.1038/s41598-019-55130-w, published online 12 December 2019

The original version of this Article contained a typographical error in the Abstract.

“Although feeder-free hPSC-maintenance culture was suitable for cell therapy, feeder-free hPSC-derived aggregates tended to collapse during 3D-xdifferentiation culture.”

now reads:

“Although feeder-free hPSC-maintenance culture was suitable for cell therapy, feeder-free hPSC-derived aggregates tended to collapse during 3D-differentiation culture.”

This has now been corrected in the PDF and HTML versions of the Article.

