# Supplementary material for: Preconditioning the Initial State of Feeder-free Human Pluripotent Stem Cells Promotes Self-formation of Three-dimensional Retinal Tissue
Source: Sci Rep. 2019 Dec 12;9:18936. doi: 10.1038/s41598-019-55130-w (PMC6908610; doi:10.1038/s41598-019-55130-w)
Supplement: Supplementary file 1 — Supplementary Figures [file 41598_2019_55130_MOESM1_ESM.pdf]

Preconditioning the Initial State of Feeder-free Human Pluripotent Stem Cells Promotes Self-formation of Three-dimensional Retinal Tissue

Atsushi Kuwahara, Suguru Yamasaki, Michiko Mandai, Kenji Watari, Keizo Matsushita, Masayo Fujiwara, Yoriko Hori, Yasushi Hiramine, Daiki Nukaya, Miki Iwata, Akiyoshi Kishino, Masayo Takahashi, Yoshiki Sasai & Toru Kimura

Supplementary Figures

Figure S1. Effects of MEF addition in 3D-differentiation culture of Ff-hiPSCs.

Figure S2. Effects of bottom-shape of culture plates in 3D-differentiation culture of Ff-hiPSCs.

Figure S3. mRNA expression in preconditioned iPSCs.

Figure S4. Comparison of 3D-retina derived from Ff-hiPSCs preconditioned with SB+SAG or LDN+SAG.

Supplementary Movie

Z-stack analysis of engrafted Ff-hiPSC-derived 3D-retina in RD-nude rat. Supplemental to Figure 6.

Figure S1

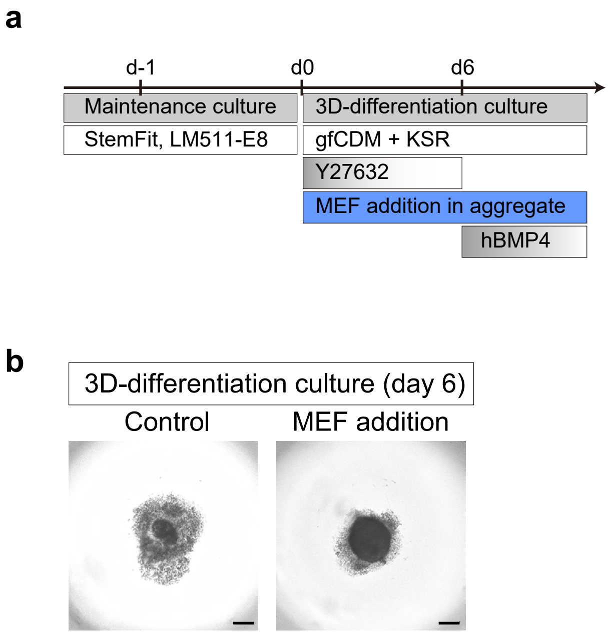

Figure S1. Effects of MEF addition in 3D-differentiation culture of Ff-hiPSCs.
(**a,b**) Ff-hiPSCs (1231A3 line) were dissociated and plated in the presence (MEF addition) or absence (Control) of MEFs. (a) Scheme. (b) Bright-field view of spheres on day 6.
Scale bars represent 200 µm in (b).
Supplementary to Figure 1.

Figure S2

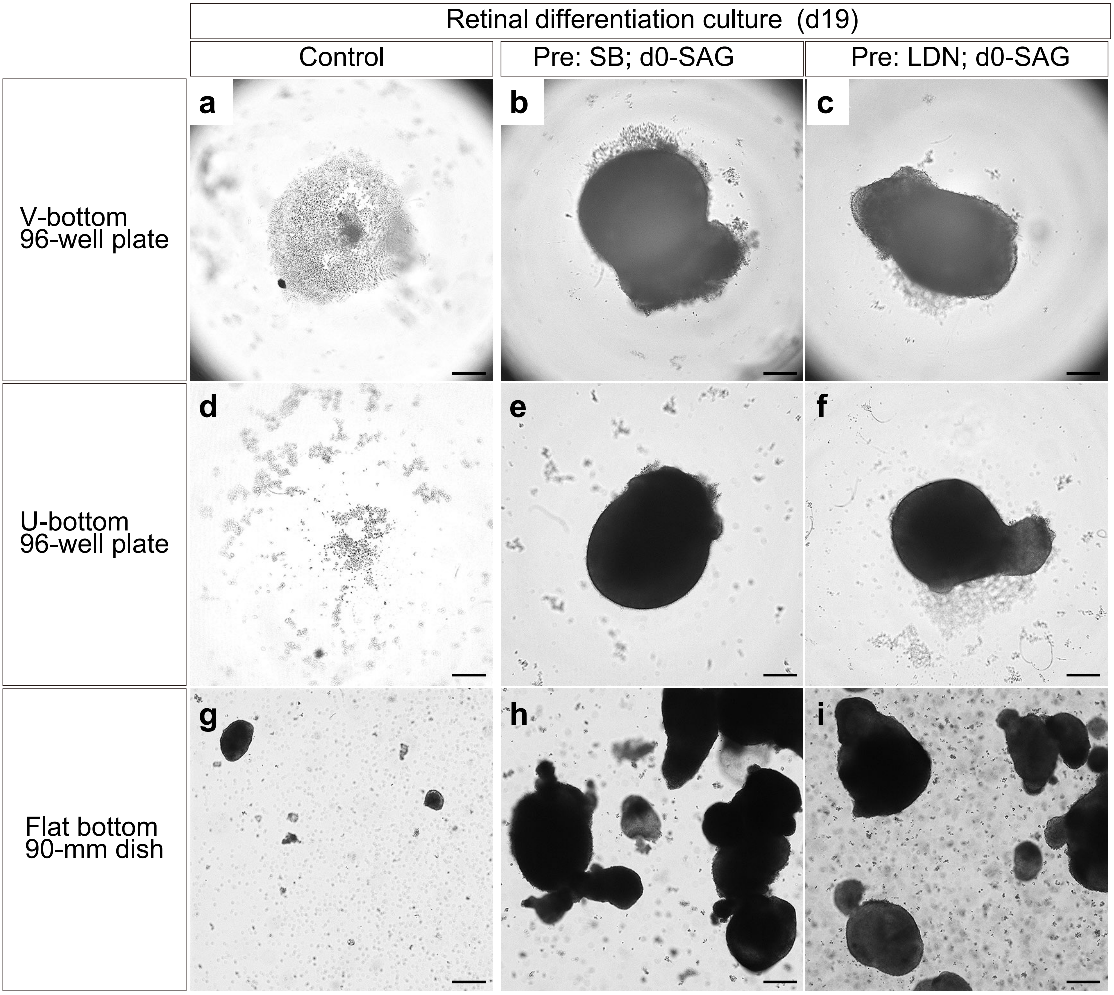

Figure S2. Effects of bottom-shape of culture plates in 3D-differentiation culture of Ff-hiPSCs. **(a–i)** Comparison of neuroepithelium-inducing efficiency among various bottom-shapes of culture plates. Ff-hiPSCs (1231A3 line) were cultured with SB (b,e,h) or LDN (c,f,i) or left untreated as control cells (a,d,g). The cells were then plated in V-bottom plates (a–c), U-bottom plates (d–f), or flat-bottom dishes (g–i). Bright-field views of spheres on day 19 are shown.
Scale bars represent 200 µm in all panels.
Supplementary to Figure 2.

Figure S3

mRNA expression in preconditioned iPSCs on day 0

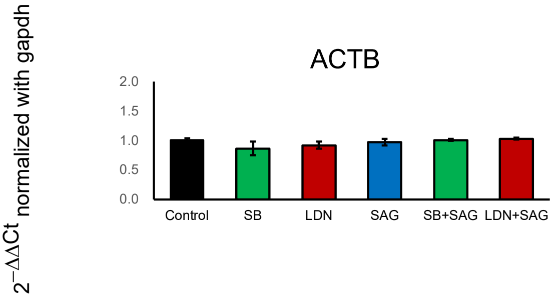

Figure S3. mRNA expression in preconditioned iPSCs.
Ff-hiPSCs (LPF11 line established with Sendai virus vectors) were treated with SB, LDN, SAG, SB+SAG and LDN+SAG for 24 h (preconditioned iPSCs) or untreated as control (Control). mRNA levels were determined by qPCR analysis. Data are represented as mean ± SEM (n = 4 experiments). *, $p < 0.05$. **, $p < 0.01$. ***, $p < 0.001$. ANOVA followed by post-hoc Tukey' s test.
Supplementary to Figure 3.

Figure S4

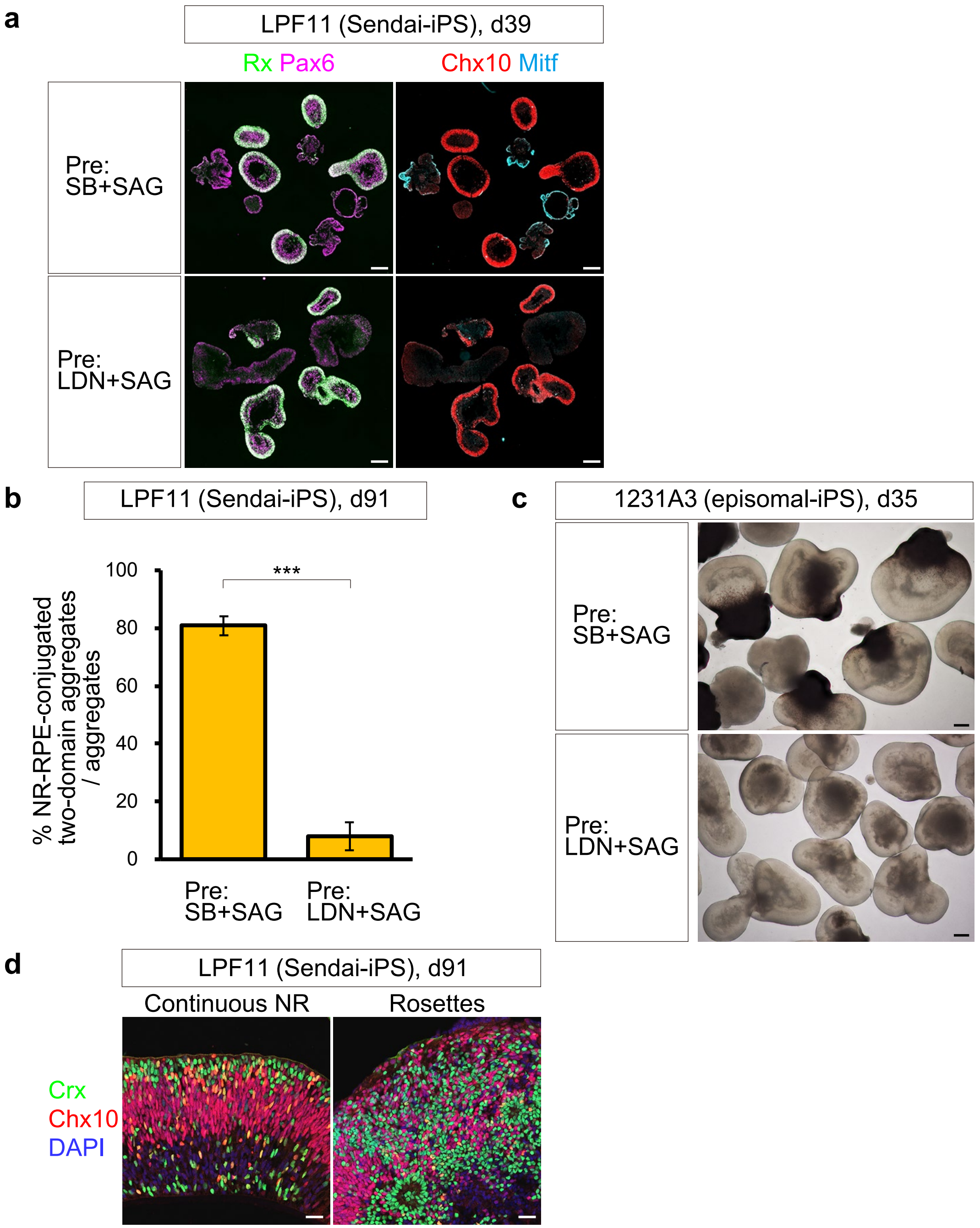

Figure S4. Comparison of 3D-retina derived from Ff-hiPSCs preconditioned with SB+SAG or LDN+SAG. **(a,b)** Ff-hiPSCs (LPF11 line) were preconditioned with SB+SAG or LDN+SAG for 1 day, treated with d0-SAG and then differentiated into 3D-retina. (a) Immunostaining for retinal markers in aggregates on day 39: Rx (green, left), Pax6 (purple, left), Chx10 (red, right), and Mitf (light blue, right). (b) Percentages of NR-RPE-conjugated two-domain aggregates on day 91. Data are represented as mean ± SEM (n = 4 experiments, with 12 aggregates per experiment). ***$p < 0.001$. Student' s t-test.
(c) Ff-hiPSCs (1231A3 line) were preconditioned with SB+SAG or LDN+SAG for 1 day and then differentiated into 3D-retina. Bright-field views are shown.
(d) Immunostaining of Ff-hiPSC (LPF11 line)-derived 3D-retinas with a continuous NR epithelium (Continuous NR) and rosette structures (Rosettes) on day 91.
Scale bars represent 200 µm in (a,c) and 20 µm in (d).
Supplementary to Figure 4.
